# Long-term patient-reported outcomes following allogeneic hematopoietic cell transplantation

**DOI:** 10.1038/s41409-025-02540-2

**Published:** 2025-02-26

**Authors:** Sina Alexandra Beer, Johanna Blättel, Kristina Reuß, Claus-Philipp Maier, Christoph Faul, Wichard Vogel, Wolfgang Bethge, Claudia Lengerke

**Affiliations:** 1 Department of Internal Medicine II, Hematology, Oncology, Clinical Immunology and Rheumatology, University Hospital Tübingen, Tübingen, Germany; 2 Department of General Pediatrics, Hematology/Oncology, Children’s University Hospital Tübingen, Tübingen, Germany

**Keywords:** Quality of life, Haematological cancer

## Abstract

Therapeutic progress has improved the overall survival of patients treated with allogeneic hematopoietic cell transplantation (alloHCT). Thus, the impact on quality of life (QoL) becomes increasingly relevant. However, QoL is not monitored regularly in clinical practice, and most trials stop QoL assessments early post-alloHCT, missing long-term dynamics. To address this knowledge gap, we conducted a cross-sectional survey of 214 adult alloHCT recipients (average age 53 y, 42.5% female, median follow-up 56 months) to evaluate QoL using patient-reported outcome measurements (PROMs), spanning a period from 30 days to over 10 years post-transplant. Participants completed the EORTC QLQ-C30 and FACT-BMT at a single follow-up timepoint to investigate QoL-related factors. Comparing long-term follow-up patients (beyond year 3, *n* = 125) with short-term follow-up patients (day 30 to month 12, *n* = 89) shows significantly better long-term QoL outcomes (*P* = 0.016). However, PROM symptom scales indicate moderate fatigue and insomnia rates in long-term survivors. Better QoL was associated with male gender, lower ECOG, RIC conditioning, no relapse, no ongoing immunosuppression and full-time work. Summarized, while we observe encouraging long-term outcomes, our data suggest that QoL recovery remain highly individual. We strongly recommend the use of PROMs to enhance our understanding of long-term survivorship post-alloHCT.

## Introduction

Over the last decades, therapeutic progress has substantially increased overall survival of patients treated with allogeneic hematopoietic cell transplantation (alloHCT) [1–3]. With prolonged survival, the impact of treatment and disease associated morbidities on the quality of life (QoL) of alloHCT recipients gains increasing relevance [4, 5]. While growing evidence highlights this problem from the patients’ perspective, QoL is often not properly monitored in routine clinical care [5–7]. Patient-reported outcome measurements (PROMs) are increasingly used in clinical trials to assess QoL and symptom burden, demonstrating greater sensitivity in these domains compared to physician assessments [8]. PROMs focus mainly on how a patient survives after a treatment, and not only on whether or how long a patient survives. However, most clinical trials stop QoL assessment within the first year after alloHCT, leaving long-term effects unexplored [9–12]. This might be due to the early positive recovery observed after alloHCT, with 80% of patients reporting good QoL and satisfactory social reintegration by day 100 post-alloHCT [13, 14]. This translates to good QoL in over 60% of patients within 1 to 4 years post-alloHCT [6]. Few studies have examined QoL outcomes 5 to 10 years post-transplant, with heterogeneous findings reporting both excellent QoL [15, 16] and persistent challenges in a substantial subset of patients [17]. These challenges are reflected in multiple PROM domains, including functional, social, emotional and cognitive functioning [18–20]. Little is known about the evolution of PROM domains over time, and evidence for QoL recovery beyond 3 years post-transplant remains sparse [21].

In this study, we evaluate QoL of alloHCT recipients using PROMs covering a post-transplant period of 10 years and beyond. The aim of this study was to explore time-dependent patterns and influencing factors on QoL in alloHCT recipients to better understand QoL dynamics. We hypothesize that:

1. QoL and symptom burden differ between short-term (up to year 1 post-transplant) and long-term follow-up ( > 3 years post-transplant).
2. Patient- (gender, age, ECOG, employment status), treatment- (conditioning regime, HLA status, immunosuppression, GvHD) and disease-related factors (remission, relapse) impact QoL outcomes.

## Patients and methods

**Fig. 1:**
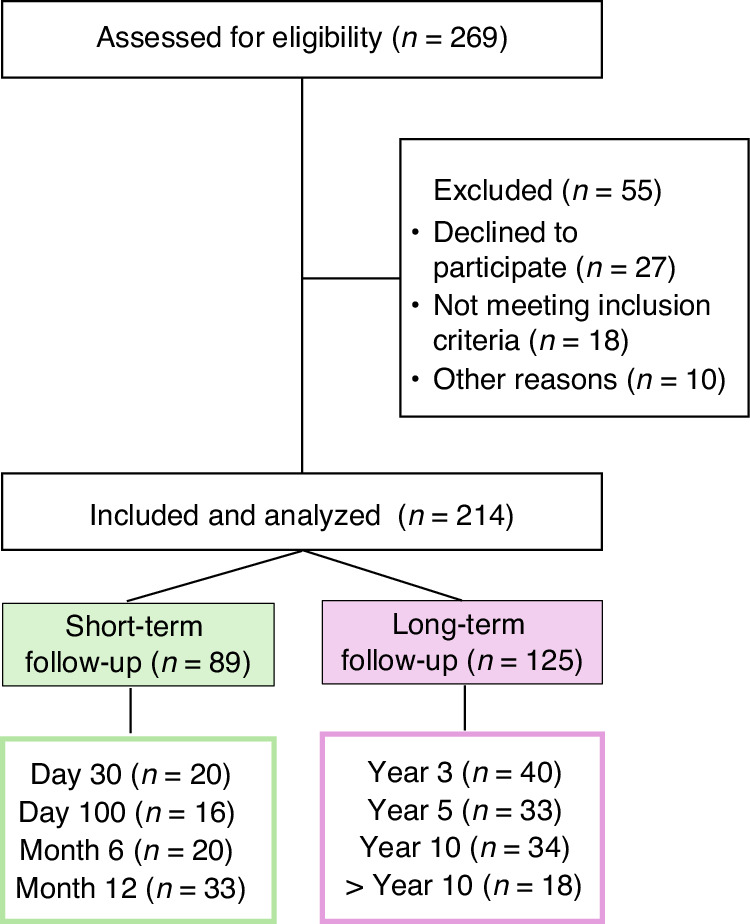
Study Flow. The composition of the study population after allogeneic hematopoietic cell transplantation (alloHCT) is shown, categorized into short-term follow-up (d30, d100, m6, m12) and long-term follow-up (y3, y5, y10, >y10).

This cross-sectional survey included adult alloHCT recipients enrolled in outpatient follow-up care from March 21st to August 1st, 2023, at the University Hospital Tübingen in Germany (Fig. 1). Patients were recruited during their face-to-face appointment and participated at a single time-point. Based on the time elapsed since alloHCT, each patient was assigned to one of eight time point cohorts: day 30 (d30), day 100 (d100), month 6 (m6), month 12 (m12), year 3 (y3), year 5 (y5), year 10 (y10) and >10 years ( > 10 y). The eight cohorts were categorized into two follow-up periods: short-term (d30 to m12) and long-term (y3 to ≥ y10). In the case of a 2nd alloHCT (*n* = 7), this determined cohort allocation. Follow-up times were calculated from the day of alloHCT (i.e., d0). Inclusion criteria were status post-alloHCT with a follow-up period ≥ 30 days, age older 18 years, written informed consent, outpatient setting, and basic knowledge of the German language. Exclusion criteria were refusal to participate, inpatient setting or no in-person visit at the transplant clinic due to other reason, reduced vigilance or disorientation (including neurodegenerative diseases, especially dementia, to ensure accurate PROMs), and insufficient language comprehension. Eligible participants completed two different PROMs, the EORTC Core Quality of Life questionnaire (EORTC QLQ-C30, version 3, open access) and the Functional Assessment of Cancer Therapy General (FACT-G, version 4, permission obtained) with the subscale for Bone Marrow Transplantation (FACT-BMT). Both PROMs were completed during a single session, once only and in-person. A short interview followed the PROMs to assess ECOG performance status, self-care, and perception of social support. In addition, patients were asked about their employment status to assess their ability to work (i.e., workability), and self-perceived infection rates during the last year. Further data was collected through electronic health records. Information on the alloHCT characteristics included transplant date, conditioning regimen (myeloablative (MAC) vs. reduced intensity conditioning (RIC), as defined by the EBMT [22]), transplant type (human leukocyte antigen (HLA) matched, mismatched or haplo-identical donors, related/family vs. unrelated, unmanipulated vs. manipulated for CD3^+^CD19^+^ depleted/TCRalphabetaCD19^+^ depleted/CD34^+^ selected), diagnosis, patients’ age and ECOG at alloHCT, the treatment response (complete remission (CR), partial remission (PR), stable disease (SD), relapse or progression), acute and chronic graft-versus-host disease (aGvHD and cGvHD, as defined by [23, 24]), bacterial/viral/fungal infections, immunosuppressive regimen and donor lymphocyte infusions (DLIs). When the term QoL is mentioned in the following, it refers to health-related QoL (HRQoL). The local ethical commission approved this study (Tübingen, no. 049/2023BO2).

### Statistical analysis

Statistical analyses were performed using Excel (Microsoft Office Professional Plus v. 2312), IBM® SPSS Statistics 29.0 and RStudio (Version 2024.04.0). A sample size calculation was performed (RStudio, pwr package) using the following parameters: Cohen’s d = 0.4–0.5, α = 0.05, power = 0.8, and a two-sided alternative hypothesis. The required sample size was determined to be *n* = 64–99 per follow-up period, i.e. 198 participants in total. Patient, symptom, and therapy characteristics were reported as frequencies or categorical variables. Categorical data were compared using the Chi-square test or Fisher’s exact test. For continuous variables, T-tests were used for two-group comparisons with normally distributed data (assessed by Shapiro-Wilk); otherwise, the Mann-Whitney U test was applied. For paired data (e.g., ECOG), the Wilcoxon signed-rank test was used. We performed ANOVA or Welch’s ANOVA to investigate differences in >2 groups, with multiple comparisons adjusted using a strict Bonferroni correction (corrected significance threshold: *P* = 0.000216, i.e., 0.05 / 231). Effect sizes were rated as follows: Eta-squared (η²) 0.01 (small effect), 0.06 (medium effect), and 0.14 (large effect). Correlation analyses were performed using Pearson and Spearman’s ρ. All significance tests were two-sided, and *P*-values < 0.05 was considered statistically significant.

The EORTC QLQ-C30 v.3 includes 30 single- and multi-items designed as 4-point Likert-scale, which is converted into a standardized score for each parameter (0–100%) through the calculation of a raw score and subsequent linear transformation according to the official scoring manual [25]. Items 29 and 30 are critical as they represent the global health status (GHS). The FACT-G is a 27-item questionnaire designed as 5-point Likert-scale. The BMT module supplements another 23 specific questions, of which 10 are included in the overall evaluation according to the scoring manual [26]. Each subscale ranges from 24–40 points, which results in a total score of 108 (FACT-G) and 148 points (FACT-BMT), respectively. Higher scores on QoL, well-being and functional subscales indicate better QoL, whereas higher scores on symptom scales reflect greater burden.

## Results

### Study population and follow-up

The study population is illustrated in Fig. 1, with detailed patient and therapy characteristics presented in Table 1. We surveyed a total of 214 patients who underwent alloHCT between 1998 and 2023, with a median follow-up of 56 months. Of 214 patients, *n* = 89 were assigned to the short-term follow-up group (d30 to m12), and *n* = 125 to the long-term group (≥ y3). *N* = 19 received alloHCT before 2010, including 10 treated between 1998 and 2005. The median ECOG at alloHCT was 0. At study enrollment, it was 1 for short-term and 0 for long-term follow-up patients. Remission status was documented as CR in 88.3% (*n* = 189), PR/SD in 1.4% (*n* = 3), and progression in 8.4% (*n* = 18) of cases. In total, 19.6% (*n* = 42) experienced a relapse following their alloHCT, with an average time to relapse of 32 months (median 21, range 0–182). Acute and chronic GvHD were observed in 43.9% (*n* = 94) and 44.9% (*n* = 96) of patients, respectively. For details on severity and manifestation, see Table 1. Immunosuppressive medication was being taken by 38.3% (*n* = 82) of patients at study enrollment, with highest rates at d30 and d100 (> 90%), then decreased to 40% (m6 and m12) and 20% (≥ y3). Most patients on immunosuppressive therapy received either tacrolimus (Tac) monotherapy (53.7%, *n* = 44) or Tac-based combinations (11%, *n* = 9) (Table 1). Over the past 12 months, 72.4% (*n* = 155) had no documented or memorable infections, 19.6% (*n* = 42) experienced a single infection, and 7.9% (*n* = 17) reported recurrent infections. Notably, recurrent infections accounted for 27.8% in the >10 y cohort.

**Table 1:** Study population and Follow-up characteristics.

Characteristic	Information
Study enrollment	21.03.2023–01.08.2023
**Period of alloHCT**	24.09.1998–27.06.2023
- 2005–2010/ 1998–2005	*n* = 9 / *n* = 10
2nd alloHCT	3.3% (*n* = 7)
**Follow-up**, median	56 months
**Sample size, final (**assessed for eligibility)	*n* = 214 (*n* = 269)
- Not included	Inclusion criteria not met (*n* = 18), Declined to participate (*n* = 27), Excluded, other reasonsn (*n* = 10)
**Age at alloHCT**, mean (median, range)	53 (56, 17–74)
**Gender**	Female 42.5% (*n* = 91)
Indication alloHCT	Acute leukemia: 58.4% (*n* = 125), MPN: 13.1% (*n* = 28), MDS: 9.3% (*n* = 20), Chronic leukemia: 8.9% (*n* = 19), Lymphoma/myeloma: 7.0% (*n* = 15), Other: 3.3% (*n* = 7)
Donor	Unrelated: 71.0% (*n* = 152), Related/Family: 29.0% (*n* = 62)
HLA status	Matched: 76.2% (*n* = 163), Mismatched: 13.6% (*n* = 29), Haplo-identical: 8.4% (*n* = 18), Missing information: 1.8% (*n* = 4)
Stem cell source	Peripheral blood: 89.3% (*n* = 191), Bone marrow: 4.7% (*n* = 10), unknown: *n* = 13
Graft composition (CD34+ cell count / kg), mean (range)	7.0 × 10^6 (0.87–16.7 × 10^6)
Conditioning regimen	MAC: 22.0% (*n* = 47), RIC: 78.0% (*n* = 167)
**ECOG, baseline** (at alloHCT), median (range)	0 (0–2)
- distributions	0: 76.6% (*n* = 164), 1: 11.7% (*n* = 25), 2: 0.5% (*n* = 1), unknown: 11.2% (*n* = 24)
**ECOG, follow-up** (study enrollment, median)	
- short-term follow-up (d30, d100, m6, m12)	ECOG = 1
- long-term follow-up (y3, y5, y10, >y10)	ECOG = 0
Remission rates (study enrollment)	CR: 88.3% (*n* = 189), PR/SD: 1.4% (*n* = 3), Progress: 8.4% (*n* = 18)
Minimal residual disease (MRD) diagnostics	62.60%
**Relapses**, total number	19.6% (*n* = 42)
- Time to relapse, mean (median, range)	31.9 months (21, 0–182)
- 2nd relapse	1.3% (*n* = 3)
DLI application	19.6% (*n* = 42 patients)
**Immunosuppressive medication**, rates	
- overall rate (*n* = 214)	38.3% (*n* = 82)
- d30 / d100 / m6 & m12 / > y3	95% / 93.8% / 40% / 20%
Immunosuppressive medication, type	Tacrolimus (Tac) monotherapy: 53.7% (*n* = 44), Tac-based combination: 11% (*n* = 9) Prednisolone monotherapy: 14.6% (*n* = 12), Ruxolitinib: 6% (*n* = 5), Other: 14.6% (*n* = 12)
**Acute GvHD**, total number	43.9% (*n* = 94)
Acute GvHD, severity	Grade I: 52.5%, Grade II / III: 47.5%
Acute GvHD, manifestation	Skin: 70.2% (*n* = 66), Gastrointestinal: 13.8% (*n* = 13) Other: 15.9% (*n* = 15), Multiple sites: 28.0% (*n* = 27)
**Chronic GvHD**, total number	44.9% (*n* = 96)
Chronic GvHD, severity	Mild: 52%, Moderate/severe: 48%
Chronic GvHD, manifestation	Skin: 55.2% (*n* = 53), Multiple sites: 37.4% (*n* = 39)
**Infection History** (past 12 months)	None: 72.4% (*n* = 155), Single episode: 19.6% (*n* = 42), Recurrent episodes: 7.9% (*n* = 17)

*alloHCT* Allogeneic Hematopoietic Cell Transplantation, *MPN* Myeloproliferative Neoplasm, *MDS* Myelodysplastic Syndrome, *MAC* Myeloablative Conditioning, *RIC* Reduced-Intensity Conditioning, *CR* Complete Remission, *PR/SD* Partial Remission/Stable Disease, *GvHD* Graft-versus-Host Disease.

### Quality of life

**Fig. 2:**
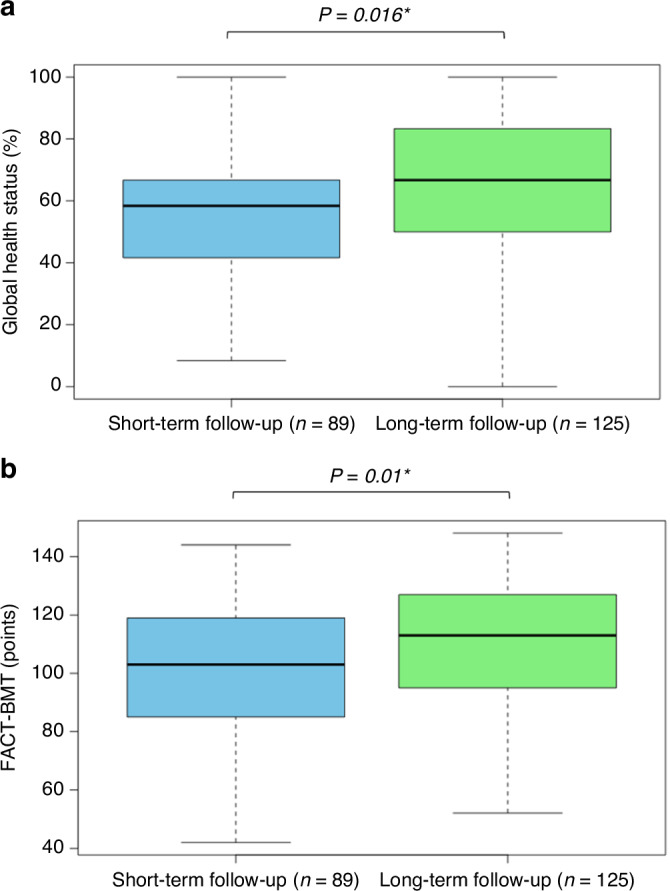
Quality of life outcomes. Quality of life (QoL) outcomes using patient-reported outcome measurements (PROMs) are compared between short-term follow-up (blue; d30, d100, m6, m12) and long-term follow-up (green; y3, y5, y10, >y10) after allogeneic hematopoietic cell transplantation (alloHCT). **a**: Global Health Status (GHS) from the EORTC QLQ-C30. **b**: FACT-BMT. Each box plot illustrates the median (central line), interquartile range (box limits), and the overall range excluding outliers (whiskers). Higher values indicate better QoL. *P*-values above the boxplots show group comparisons, with * indicating significance. X-axis: Follow-up group after alloHCT. Y-axis: Average PROM values in % (GHS) or points (FACT).

Before analyzing the PROM data, we ensured homogeneity of variance and <2% missing value rates (EORTC: 0.5%, FACT: 1.6%). As the PROM results were not normally distributed, non-parametric tests were used subsequently. In general, GHS, FACT-G, and FACT-BMT results showed strong positive correlations (*r* > 0.8, *P* < 0.01). Regarding the entire study population (n = 214), average values for GHS, FACT-G and FACT-BMT were 61.32% (SD 23.5, range 0–100), 78.17 points (SD 18.1, range 31–108), and 106.08 points (SD 24.3, range 42–148), respectively (Table 2). The long-term group (*n* = 125) showed significantly better QoL than the short-term group (n = 89) regarding GHS (57.3% vs. 64.2%, *P* = 0.016) and FACT-BMT (101.2 vs. 109.6, *P* = 0.01) (Fig. 2). To further understand QoL outcomes, we analyzed patients without relapse (*n* = 172) and with relapse history (n = 42) separately. The population without relapse reported better PROM results (e.g., GHS: 63.4 vs. 52.9%, *P* = 0.007, Table 2), particularly from follow-up y3 onward, aligning with the median time to relapse. In keeping with this, the long-term to short-term QoL difference was more pronounced when excluding patients with relapse, resulting in n = 80 short-term and n = 92 long-term cases (GHS: 58.3% vs. 67.8%; FACT-BMT: 101.9 vs. 112.8 points, both *P* = 0.002, Table 2). At the individual cohort level, patients in the m12 (*n* = 33) and y3 (*n* = 40) cohorts reported worse QoL outcomes compared to those in the d100 (n = 16) and m6 (*n* = 20). Due to the cross-sectional design, direct comparisons using inferential statistics are underpowered and challenging to interpret, therefore not reported. The >10 y survivors reported slightly worse QoL outcomes compared to the y10 cohort (y10 vs. >y10: GHS 73.4 vs. 67.2; FACT-BMT 121.1 vs. 114.8). PROM results for each individual cohort are visualized in Fig. S1.

**Table 2:** PROM results for each individual cohort (1–8), the overall study population, relapse population, and short-term vs. long-term follow-up groups.

Cohort	*n*	GHS	FACT-G	FACT-BMT	*n*	GHS	FACT-G	FACT-BMT
		mean, % (SD)	mean, pts. (SD)			mean, % (SD)	mean, pts. (SD)	
		Excl. relapse population				Incl. relapse population		
Cohort 1 (d30)	20	50.4 (21.8)	73.3 (14.9)	99.9 (21.2)	20	50.4 (21.9)	73.3 (15.0)	99.9 (21.2)
Cohort 2 (d100)	13	61.2 (21.4)	75.2 (18.6)	100.8 (25.3)	16	60.2 (22.1)	75.5 (18.7)	101.5 (24.9)
Cohort 3 (m6)	18	67.4 (18.4)	81.6 (17.2)	111.9 (22.8)	20	66.9 (18.0)	81.3 (16.4)	111.2 (21.8)
Cohort 4 (m12)	29	56.9 (23.7)	72.4 (20.7)	97.6 (27.3)	33	54.3 (23.4)	71.3 (20.5)	95.7 (27.2)
Cohort 5 (y3)	28	59.9 (28.4)	74.5 (21.1)	101.1 (27.9)	40	60.3 (25.8)	74.2 (19.6)	100.9 (25.9)
Cohort 6 (y5)	21	71.0 (23.1)	84.4 (14.6)	115.7 (18.6)	33	63.6 (27.1)	78.8 (17.4)	107.8 (22.5)
Cohort 7 (y10)	28	73.4 (19.3)	89.6 (14.6)	121.1 (20.0)	34	69.7 (21.1)	88.6 (15.1)	119.6 (20.2)
Cohort 8 (>y10)	15	67.2 (19.0)	84.5 (13.2)	114.8 (18.0)	18	63.4 (19.4)	83.0 (13.4)	113.1 (18.0)
Overall	172	63.4	79.3	107.7	214	61.3	78.2	106.1
Relapse population	42	52.9	73.4	99.4				
***P value*** *no relapse vs. relapse*		0.007*	0.034*	0.022*				
Short term	80	58.3	75.2	101.9	89	57.3	74.7	101.2
Long term	92	67.8	83	112.8	125	64.2	80.6	109.6
***P value*** *short vs. long*		0.002*	0.003*	0.002*		0.016*	0.015*	0.01*

Sample sizes (*n*) for each average QoL outcome are provided. Two-group comparisons were conducted using the Mann-Whitney *U* test, with two-sided *P*-values indicated and significant results marked with an asterisk (*). *GHS* Global Health Status, *SD* standard deviation.

**Fig. 3:**
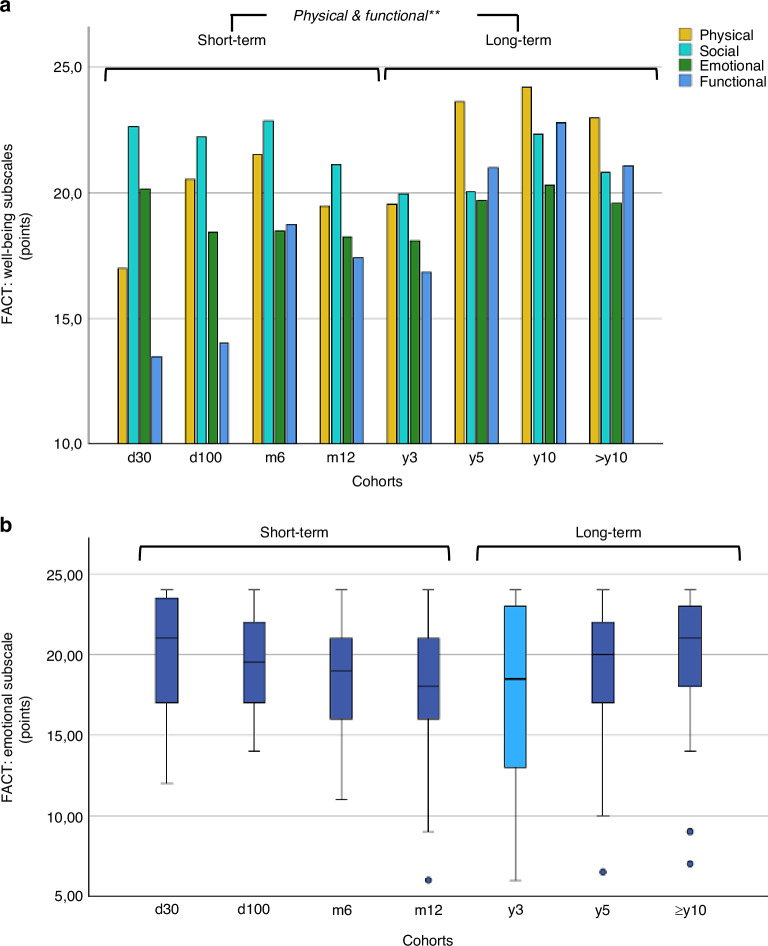
Well-being subscale results. Well-being subscale results over a 10-year follow-up period following allogeneic hematopoietic cell transplantation (alloHCT). Each box plot illustrates the median (central line), interquartile range (box limits), and the overall range excluding outliers (whiskers). Outliers are marked with circles. Higher values indicate better well-being. **a** FACT subscales showing physical (yellow), social (turquoise), emotional (green), and functional (blue) well-being. Long-term vs. short-term follow-up with significant differences in physical and functional well-being (*P* = < 0.01**). **b** Distribution of the FACT subscale “emotional” per individual cohort. X-axis: Time points (cohorts) post-alloHCT; day (d), month (m), year (y). Y-axis: Average values of the PROM subscales in points.

In a next step, we examined the PROM functional and well-being subscales (Table 3). Average values for patients with and without relapse, are listed in Table S1. Across all eight cohorts, ANOVA revealed significant variance in physical (EORTC: *P* = 0.011, FACT: *P* = 0.004), role (EORTC: *P* < 0.001), social (EORTC: *P* = 0.018), and functional (FACT: *P* < 0.001) well-being (all *P*-values Bonferroni corrected) (Fig. 3a). All above-mentioned subscales showed significantly better outcomes in long-term vs. short-term follow-up (Table 3). Based on the documented effect sizes, role (η^2^ = 0.153) and functional well-being (η^2^ = 0.166) appeared to have the greatest influence in the overall model. Cognitive and emotional functioning were reported to a similar extent by all participants. Notably, cognitive functioning is the only subscale where the relapse cohort showed better results (Table S1), although the difference was not significant (*P* = 0.724). Emotional functioning presented notably wide inter-individual ranges, particularly in y3 (Fig. 3b).

**Table 3:** PROM subscale results for short-term vs. long-term follow-up groups.

PROM subscale	Unit	Short-Term (*n* = 89)	Long-Term (*n* = 125)	*P*-value *short vs. long*
**Functioning subscales (EORTC-QLQ30)**				
Physical	mean, %	63.6	73.5	0.006*
Role		46.1	61.3	0.001*
Emotional		66.8	69.2	0.316
Cognitive		70.8	72.2	0.418
Social		51.9	64.2	0.003*
**Well-being subscales (FACT-G/BMT)**				
Physical	mean, points	18.9	21.8	0.001*
Social		21.6	20.4	0.068
Emotional		18.5	18.8	0.467
Functional		15.7	19.7	< 0.001*
**Symptom subscales (EORTC-QLQ30)**				
Fatigue	mean, %	53.5	43	0.013*
Nausea		11.2	7.6	0.097
Pain		30.5	25.5	0.184
Dyspnea		35.6	33.2	0.645
Insomnia		35.9	29.9	0.208
Appetite		29.6	15.2	0.002*
Constipation		8.2S	12.9	0.069
Diarrhea		20.2	15.5	0.121
Financial difficulties		29.2	20.1	0.036*

Two-group comparisons were conducted using the Mann-Whitney *U* test, with two-sided *P*-values indicated and significant results marked with an asterisk (*).

**Fig. 4:**
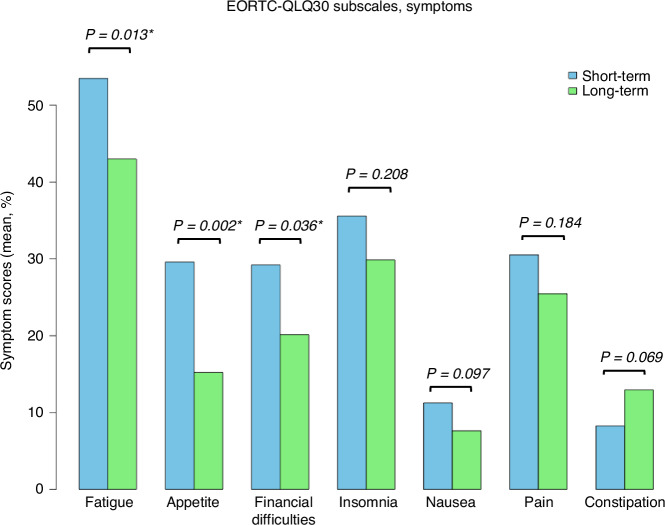
Symptom subscale results. Seven symptoms from the EORTC QLQ-C30 are displayed. For each symptom, the average score (%) is shown as a bar plot for both short-term (blue) and long-term (green) follow-up. Higher values indicate greater symptom burden. *P*-values for two-group comparisons are shown above each symptom. Fatigue, appetite loss, and financial difficulties are significantly different (*), with a lower symptom burden in the long-term follow-up group.

Lastly, we investigated the symptom scales in the EORTC QLQ-30 (Table 3). The short-term follow-up group reported a higher symptom burden in all subscales, except for constipation, which was higher in the long-term group (Fig. 4). Significant differences were observed for fatigue (*P* = 0.013), appetite loss (*P* = 0.002), and financial difficulties (*P* = 0.036), with fewer symptoms in the long run (Table 3). Insomnia, pain, nausea and diarrhea showed no significant variation across time points. When examine the individual cohorts, again, patients in the m12 and y3 cohorts reported more symptoms (e.g., fatigue, pain, and dyspnea) compared to the other cohorts (Table S1). Patients with relapse history reported significantly more fatigue (57.1 vs. 44.8 pts.) and dyspnea (48.0 vs. 30.8 pts.) compared to the no-relapse cohort (*P* = 0.02 and *P* = 0.004, respectively). Similarly, pain was more often stated in patients with relapse (35.3 vs. 25.7 pts.), though this difference was not statistically significant (*P* = 0.088) (Table S1).

### Quality of life, influencing parameters

We investigated the impact of patient- (gender, age, ECOG, employment status), treatment- (conditioning regime, HLA status, immunosuppression, GvHD) and disease-related factors (remission, relapse) on QoL outcomes. The employment status was known for 93.0% (*n* = 201), with 24.8% (*n* = 60) were on sick leave or unemployed, 40.7% (*n* = 87) were retired, 22.4% (*n* = 48) had returned to full-time work, and 2.8% (*n* = 6) were self-employed. The maximum proportion of full-time workers (53.2%, *n* = 114) and retirees (64.3%, *n* = 137) was recorded in y10 and >y10, respectively. Sick leaves nearly diminished over time (y10: 6.3%, >y10: 0%). Most patients (91.1%) stated good social support, 100% support was documented at d30 and d100.

For clarity, we report *P*-values based on GHS below; *P*-values for each PROM are provided in Table S2. Associated with better QoL outcomes were male gender (*P* = 0.013), better ECOG (*P* = 0.001), RIC conditioning regime (*P* = 0.03), no history of relapse (*P* = 0.002), no ongoing immunosuppression (*P* < 0.001) and full-time work or self-employment (*P* < 0.001) (Table S2). Regarding immunosuppression, its impact on QoL was significant for the presence or absence of immunosuppressive treatment, but not for the type of immunosuppression. Tac-based regimens showed a slight trend towards better QoL compared to other regimens (Fig. S2). No significant association was seen for age (*P* = 0.247), HLA status (*P* = 0.261), and acute (*P* = 0.334) or chronic GvHD (*P* = 0.550). Disease remission exhibited a trend toward significance in FACT-BMT (*P* = 0.077) but not in GHS (*P* = 0.244). Additional non-parametric correlation analyses for aGvHD and cGvHD occurrence with QoL outcomes likewise confirmed no significant associations (*P* = 0.149 and *P* = 0.282, respectively). Acute and chronic GvHD did furthermore not correlate with DLI application (*P* = 0.419 and 0.444) or administered CD34+ stem cell counts (*P* = 0.836 and 0.378). Given that the study spans 25 years of alloHCT, we examined the influence of the transplantation date on QoL outcomes. Although there was a significant correlation (rho = 0.25, *P* < 0.001), indicating a weak relationship, the visualization shows no clear pattern (Fig. S3).

## Discussion

In this study, we conducted a cross-sectional evaluation of QoL and symptom burden among alloHCT recipients, spanning a follow-up period from 30 days to over 10 years post-transplant. The key finding was that the majority of patients achieved sustained good QoL levels beyond year 3 post-transplant. The long-term follow-up group (*n* = 125) showed significantly better QoL outcomes compared to the short-term group (*n* = 89, GHS *P* = 0.016, FACT *P* = 0.01), with the difference becoming more pronounced when excluding patients with a relapse history (*n* = 42). Contextualizing this finding, our good long-term QoL results closely align with the outcomes reported by Lee et al. [27] and others [15, 16]. Fortunately, the QoL outcomes of the y10 cohort are comparable to those of the general population (GHS 69.7% vs. 67.0% [28], FACT-G 88.6 vs. 85.9/82.3 pts. general population/cancer patients [29, 30]). Nevertheless, PROM symptom scales indicated moderate fatigue and insomnia rates in long-term survivors, although these rates were lower than those in the short-term group. On the other hand, our data could not fully support the excellent QoL outcomes reported in years 1–4 [6], as worse overall and subscale scores were observed in the m12 and y3 cohorts. While this is cross-sectional data and individual cohort sizes are rather small, the robustness of these findings and their potential generalizability warrant careful consideration. QoL is influenced by various factors, such as patients’ personality, social and financial issues [31]. In keeping with this, we observed significant inter-individual variation in PROM subscales, highlighting role and functional well-being as key factors in overall QoL. Despite the cross-sectional design, distinct needs are already evident within our study population.

In addition to individual factors and personality as explanations for inter-individual variation, we believe that our observations could also be plausible in a longitudinal trial setting. The rather positive QoL reports in our d100 and m6 cohorts may reflect a compensation for the emotionally and physically demanding diagnosis and treatment period. However, after the first year, recipients likely confront the more challenging reality. In our study population, a maximum of only 53.2% went back to full-time work, which aligns with proportions reported in comparable studies [32]. Other studies in the field confirm the challenges related to workability and financial burden faced by patients two years post-transplant [17, 32]. As also supported by the literature, we validated the impact of ECOG and conditioning regime [33, 34], as well as a relapse history on QoL outcomes. Patients with a relapse history reported significantly more fatigue and dyspnoea, suggesting that using PROMs for early relapse detection could be potentially useful. Additionally, the median time to relapse of 20 months may explain the poorer QoL outcomes seen in m12 and y3 patients. Controversially, we could not find statistical evidence for a relation between QoL and GvHD occurrence, which contrasts previous findings [6, 35]. This may be attributed foremost to the focus on an outpatient setting and the resulting selection bias, likely involving milder GvHD cases, as well as the small cohort sizes. The rates of acute (43.9%) and chronic (44.9%) GvHD in our population were relatively low compared to rates in the literature [36–38]. However, we observed a significant influence of ongoing immunosuppression on QoL scores. Finally, considering the 25-year period (1998–2023) and changes in alloHCT procedures, we ruled out a major impact on QoL outcomes. Even so, >10-year survivors reported slightly worse QoL, suggesting this remains possible.

Looking at our PROM instruments, the EORTC-QLQ30 and FACT-G/BMT have proven to be sufficient tools to inquire QoL with respect to patients’ acceptance as well as comparability with previously published data. The scores correlated strongly amongst each other (*r* > 0.8), underscoring their homogeneity. FACT-G/BMT appeared slightly more sensitive to detect QoL changes on the long run, however, the EORTC QLQ30 provides valuable information on symptom burden. While the cross-sectional study design provided a snapshot of patients’ clinical and functional status across different follow-up periods after alloHCT, it also represents the main limitation of the study. The design does not allow for conclusions about changes over time or causality. Additionally, the study lacked power for cohort-specific analysis and faced selection bias from outpatient settings. However, our study is one of few QoL studies in the alloHCT setting that extends evaluation beyond two years post-transplant and included a reasonable number of patients. It reinforces existing data, highlighting the potential for positive long-term outcomes while stressing the importance of individual variations. Follow-up care should integrate PROMs from baseline onward to identify patient needs, particularly addressing fatigue and insomnia rates, as well as the more vulnerable relapse population. Further research is needed to enhance our understanding of long-term survivorship after alloHCT, especially in the growing body of outpatient care.

## Supplementary information

Supplemental Material - Legends

Supplementary Table_S1

Supplementary_Table_S2

Figure S1

Figure S2

Figure S3

## Data Availability

For original data, please contact sina.beer@med.uni-tuebingen.de
