# Supplementary material for: Long-term patient-reported outcomes following allogeneic hematopoietic cell transplantation
Source: Bone Marrow Transplant. 2025 Feb 26;60(5):617–24. doi: 10.1038/s41409-025-02540-2 (PMC12061752; doi:10.1038/s41409-025-02540-2)
Supplement: Supplementary file 1 — Supplemental Material - Legends [file 41409_2025_2540_MOESM1_ESM.docx]

Supplementary Material – Legends

| **Supplementary Table S1:** PROM subscale analysis. Average values with standard deviation (SD) are shown for patients with and without relapse history, as well as for each individual time-point cohort. |
| --- |
| **Supplementary Table S2:** Quality of life differences by patient, disease, and treatment characteristics. Significant results are marked with *. Statistical analysis was performed using the Mann-Whitney U test. *P*-values are two-sided. |
| **Figure S1:** PROM results for each individual cohort over a 10-year period following allogeneic hematopoietic cell transplantation (alloHCT). Top graph: Global health status (GHS) of the EORTC-QLQ30, Bottom graph: FACT-BMT. Average values per cohort are represented by diamonds, with error bars indicating the 95% confidence intervals (CI). Higher values indicate better QoL. X-axis: Time points (cohorts) after alloHCT. Y-axis: Average values of the respective PROMs in % (GHS) or points (FACT). |
| **Figure S2:** Mean Global Health Status (GHS) by immunosuppression regimen. Patients were categorized into four groups based on their immunosuppressive treatment: no immunosuppression (n=132), tacrolimus-based regimens (n=53), prednisolone (n=12), and other regimens (n=17). Error bars indicate standard errors of the mean. |
| **Figure S3:** Scatter plot of FACT-BMT scores (in points) versus days since alloHCT, showing a weak positive correlation (Spearman’s rho = 0.25, P < 0.001). The red line represents the smoothed trend with a 95% confidence interval (shaded area). The visualization does not suggest a clear linear pattern. |
