# Supplementary figures and images for: Long-term patient-reported outcomes following allogeneic hematopoietic cell transplantation
Source: Bone Marrow Transplant. 2025 Feb 26;60(5):617–24. doi: 10.1038/s41409-025-02540-2 (PMC12061752; doi:10.1038/s41409-025-02540-2)

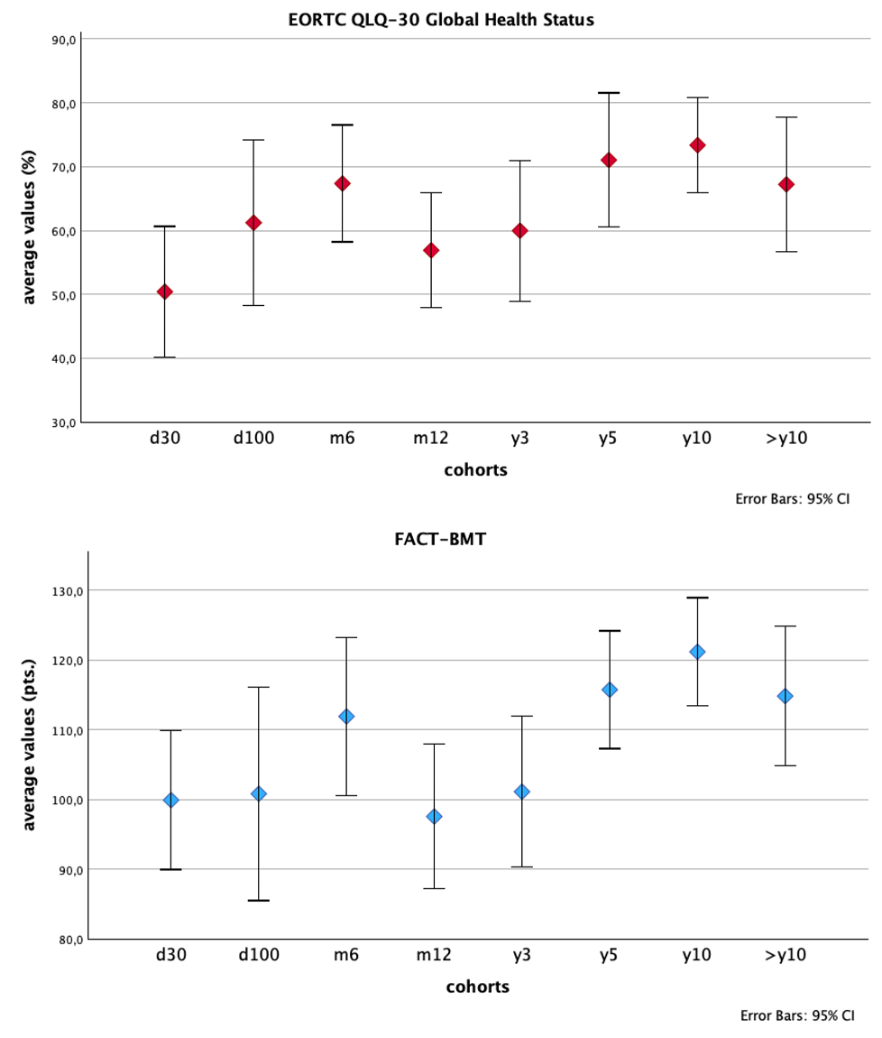

Supplement: Supplementary file 4 — Figure S1 [file 41409_2025_2540_MOESM4_ESM.png]

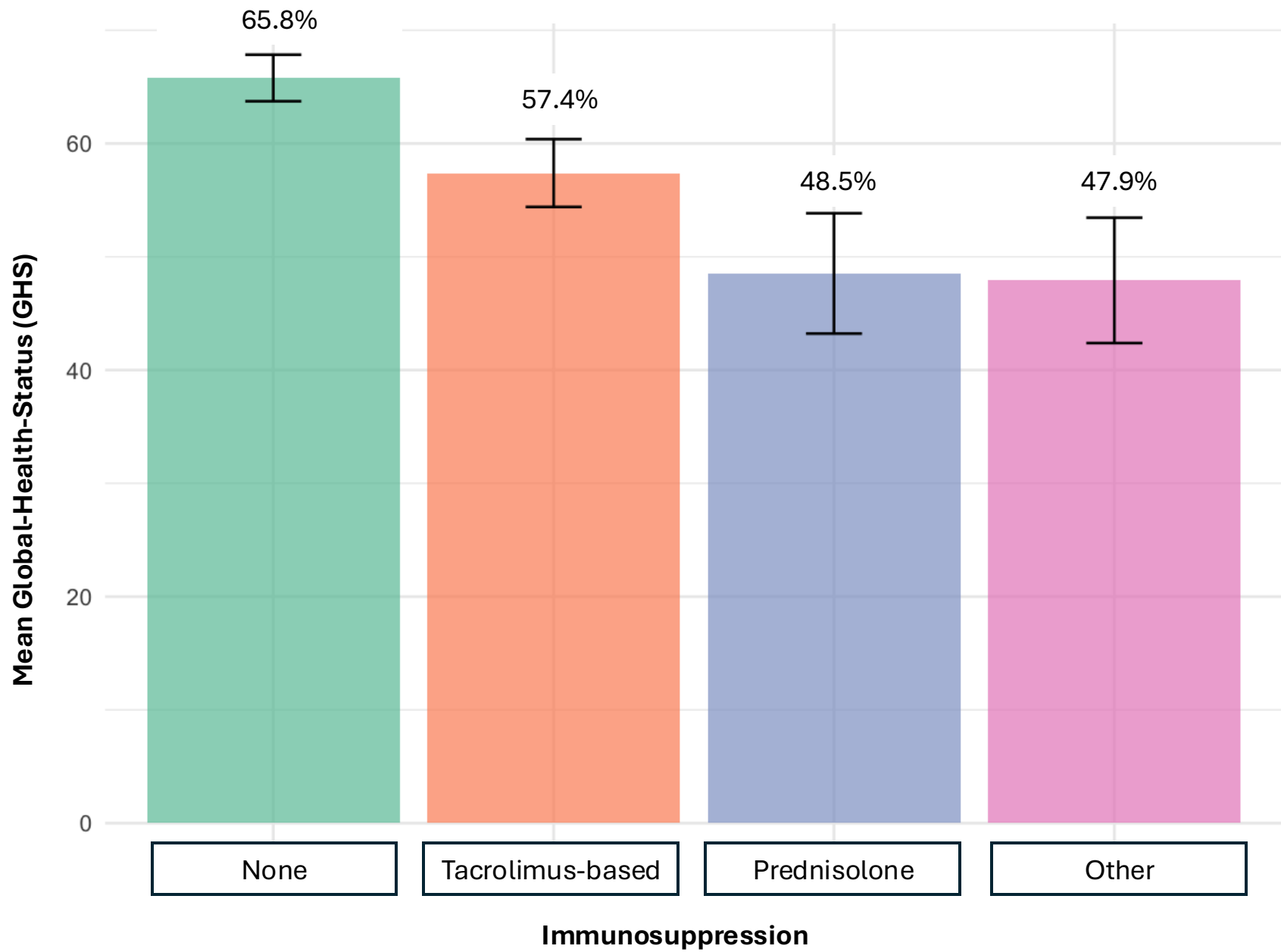

Supplement: Supplementary file 5 — Figure S2 [file 41409_2025_2540_MOESM5_ESM.pdf]

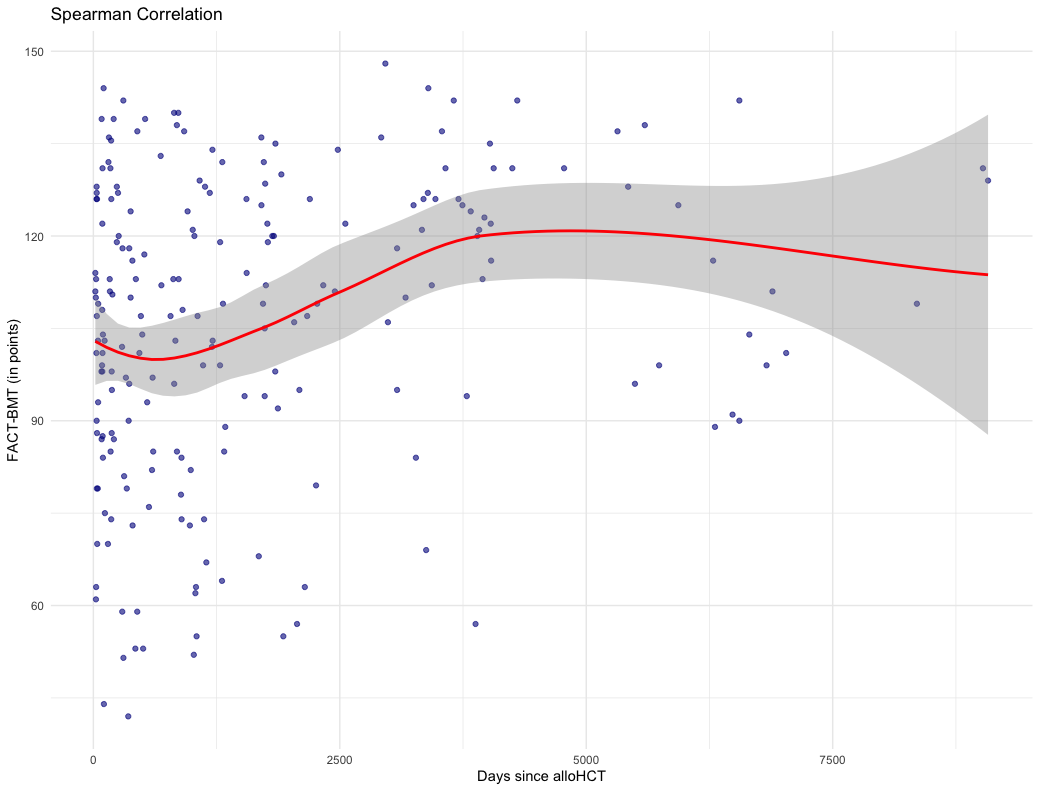

Supplement: Supplementary file 6 — Figure S3 [file 41409_2025_2540_MOESM6_ESM.png]
